# Endometrial Decidualization: The Primary Driver of Pregnancy Health

**DOI:** 10.3390/ijms21114092

**Published:** 2020-06-08

**Authors:** Shu-Wing Ng, Gabriella A. Norwitz, Mihaela Pavlicev, Tamara Tilburgs, Carlos Simón, Errol R. Norwitz

**Affiliations:** 1 Department of Obstetrics & Gynecology, Tufts University School of Medicine, Boston, MA 02111, USA; 2 Mother Infant Research Institute, Tufts Medical Center, Boston, MA 02111, USA; 3 School of Public Health, University of Michigan, Ann Arbor, MI 48109, USA; gnorwitz@umich.edu; 4 Department of Theoretical Biology, University of Vienna, 1010 Vienna, Austria; mihaela.pavlicev@univie.ac.at; 5 Division of Immunobiology and Center for Inflammation and Tolerance, Cincinnati Children’s Hospital, Cincinnati, OH 45229, USA; tamara.tilburgs@cchmc.org; 6 Department of Pediatrics, University of Cincinnati College of Medicine, Cincinnati, OH 45229, USA; 7 Department of Obstetrics & Gynecology, Valencia University and INCLIVA, 46010 Valencia, Spain; carlos.simon@uv.es; 8 Department of Obstetrics & Gynecology, Beth Israel Deaconess Medical Center, Harvard University, Boston, MA 02215, USA; 9 Igenomix Foundation and INCLIVA, 46010 Valencia, Spain

**Keywords:** endometrium, decidualization, adverse pregnancy outcome, preconception

## Abstract

Interventions to prevent pregnancy complications have been largely unsuccessful. We suggest this is because the foundation for a healthy pregnancy is laid prior to the establishment of the pregnancy at the time of endometrial decidualization. Humans are one of only a few mammalian viviparous species in which decidualization begins during the latter half of each menstrual cycle and is therefore independent of the conceptus. Failure to adequately prepare (decidualize) the endometrium hormonally, biochemically, and immunologically in anticipation of the approaching blastocyst—including the downregulation of genes involved in the pro- inflammatory response and resisting tissue invasion along with the increased expression of genes that promote angiogenesis, foster immune tolerance, and facilitate tissue invasion—leads to abnormal implantation/placentation and ultimately to adverse pregnancy outcome. We hypothesize, therefore, that the primary driver of pregnancy health is the quality of the soil, not the seed.

## 1. Introduction

Many complications that manifest clinically in the first trimester—such as miscarriage—or in the latter half of pregnancy—including preeclampsia, preterm birth (PTB), fetal growth restriction (FGR), and gestational diabetes (GDM)—have their origins early in gestation with abnormalities in implantation and placentation [1,2,3,4,5,6,7,8]. Despite exhaustive research and a vastly improved understanding of the molecular and cellular mechanisms responsible for implantation/placentation, interventions to prevent these complications have been largely unsuccessful. In this monograph, we suggest this is because the foundation for pregnancy health is laid down earlier than previously appreciated during the preconception period at the time of endometrial decidualization. Humans are one of only a few mammalian viviparous species in which decidualization starts during the latter half of each menstrual cycle and is therefore independent of the conceptus [9,10,11]. This implies that the health of a pregnancy is determined even before the blastocyst arrives. Once a pregnancy is established, its destiny has already been determined and it is too late to intervene effectively. Stated differently, pregnancy complications are not two-stage disorders as conventionally understood with abnormal implantation/placentation leading to clinical disease, but rather three-stage disorders starting with abnormal endometrial decidualization that predates the arrival of the blastocyst leading thereafter to abnormal implantation/placentation and ultimately to clinical disease [5]. We hypothesize therefore that the primary driver of pregnancy health is the quality of the soil, not the seed.

## 2. Biological Continuum of Adverse Pregnancy Outcome

While we divide pregnancy disorders into distinct categories, much of this classification is arbitrary for the purposes of description and study. Delivery at 19 weeks 6 days of gestation is defined as a miscarriage, whereas delivery one day later is a premature birth. In reality, these conditions occur along a continuum. They have common and interrelated risk factors. For example, a woman with a history of a prior unexplained PTB at 28 weeks is at increased risk of spontaneous PTB in a subsequent pregnancy, but is also at increased risk of preeclampsia and FGR in future pregnancies [4]. These disorders also have overlapping biomarkers [12]. Moreover, deficient spiral artery remodeling has been linked with a spectrum of obstetrical syndromes, including pre- eclampsia, FGR, PTB, premature rupture of membranes (PPROM), abortion, and fetal death [4,8,13]. Taken together, these observations suggest that adverse pregnancy events occur along a biological continuum and likely have a common underlying pathophysiology.

## 3. Implantation and Placentation

Implantation is critical to survival of a species, but this process in humans has a surprisingly high failure rate. Maximal fecundity (the likelihood of getting pregnant each cycle) peaks at 30% [2,14]. Only 50% of conceptions advance beyond 20 weeks of gestation and, of all unsuccessful pregnancies, 75% represent a failure of implantation [2,14,15,16]. Even among eutherian (placental) mammals, humans are unique. Among other features, human pregnancy has the most invasive type of placentation (hemochorial), early recognition of the fetal allograft by the maternal immune system, and a long gestational length [9,11].

The factors regulating implantation have been reviewed in detail elsewhere [2,7,17]. Briefly, as in other mammals, human implantation likely involves three steps: (i) apposition (initial adhesion, which is unstable), (ii) attachment (stable adhesion), and (iii) invasion, which occurs in two phases or waves. The ‘first wave’ of trophoblast invasion occurs between days 7 and 10 post-conception, starting shortly after the blastocyst hatches out of the zona pellucida. During this time, the blastocyst actively invades the tissues of the uterus. By day 10 postconception, the blastocyst is completely buried within the endometrial lining. For the next few weeks, the placenta is not yet hemochorial [18] and the blastocyst is fed by secretions from the endometrial glands (histiotrophic support) under conditions that are both hypoxic and hypoglycemic. Indeed, high levels of oxygen or glucose at this stage will damage the developing embryo. At 8–10 weeks of gestation, the placental extravillous cytotrophoblast cells (EVCTs) change their adhesion molecule expression and stream out of the placental villi to invade the full thickness of the decidualized endometrium (decidua) and the inner third of the myometrium. These cells invade the maternal spiral arteries, attracted in part by the high oxygen tension [19] and by active recruitment by uterine natural killer (uNK) cells and macrophages [8,17,20], and remodel these vessels by destroying the muscle layer and replacing the endothelial lining with a pseudo-endothelium of fetal origin. This process—known as the ‘second wave’ of trophoblast invasion—is usually complete by 18 weeks of gestation and is critical for the establishment of the definitive uteroplacental circulation. Interestingly, the initiation of vascular remodeling precedes the trophoblast invasion of the spiral arteries and is likely initiated by resident uNK cells [21,22]. As pregnancy progresses, the 120–140 small, tortuous maternal spiral arteries that supply each placenta need to dilate enormously to accommodate the increasing demands of the fetoplacental unit. The placenta is a high-volume, low-resistance organ. At term, almost one-fifth of the maternal cardiac output (approximately 800mL) passes through the placenta every minute. If this remodeling of the maternal spiral arteries from narrow lumen, tortuous vessels with a thick muscle layer to wide, thin-walled, funnel-shaped vessels is not adequate—a pathological hallmark known as shallow endovascular invasion [1,8]—the feto- placental unit will outgrow its blood supply, resulting in placental dysfunction and ultimately in clinical disease.

## 4. It’s the Quality of the Soil, Not the Seed

Successful implantation is the end result of a complex molecular interaction between two separate components: a viable blastocyst and an appropriately primed endometrium [2,6,23]. Both are important, but do they contribute equally to reproductive disorders? Much attention has focused on the blastocyst, and, indeed, many early miscarriages do result from karyotypic abnormalities within the blastocyst [24]. However, there is increasing evidence to suggest that appropriate priming of the soil (endometrium)—a process known as decidualization—may contribute more to reproductive disorders than the quality of the seed (embryo). Observations in support of this argument include:

- A critical period of time exists within each menstrual cycle—known as the ‘window of implantation’—in which the endometrium is maximally receptive to the blastocyst. This period is personalized and implantation outside of this 24–36 h window will result in an absolute failure to establish a pregnancy or in suboptimal implantation increasing the risk of a range of downstream adverse pregnancy events [25,26].
- In contrast to the ‘window of implantation’ in the endometrium, embryos generated by IVF can be transferred into the uterus any time between days 2 and 7 postconception [27].
- Embryos can be frozen and thawed multiple times prior to transfer.
- Oocyte donor embryos (that are entirely allogeneic) will implant successfully [28,29].
- Pregnancy outcomes appear to be better in frozen rather than fresh cycles [30]. A plausible explanation might be that the hormonal manipulations used to prepare the endometrium for existing cryopreserved embryos are more favorable to the endometrium than protocols used in fresh cycles, which are designed primarily to maximize the number of oocytes retrieved.
- Lastly, although the presence of a decidua is not an absolute requirement for implantation since the blastocyst can implant in the fallopian tube, the cervix, or even into the vasculature of the bowel in the case of extrauterine intraabdominal ectopic pregnancies, such pregnancies are rarely healthy and, if they do go past 20 weeks, have a high rate of complications.

Taken together, these data suggest that the endometrial “window of implantation” is independent of the blastocyst and that the embryo is not the rate-limiting factor for implantation, but rather the synchronization between them.

## 5. The Decidua as an Anatomically Distinct Autocrine/Paracrine Organ

**Figure 1:**
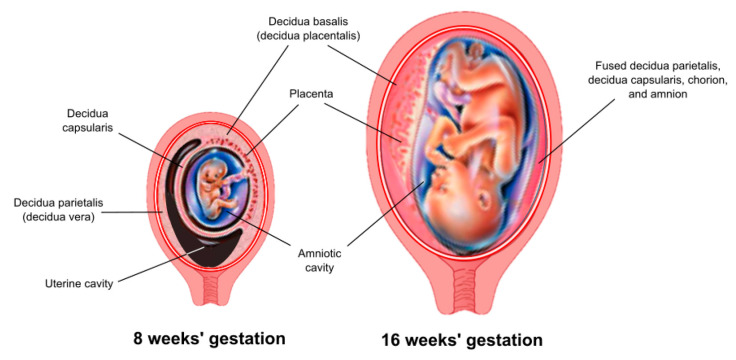
Anatomic arrangement of the decidua. Three anatomically and functionally discrete regions of the decidua are defined based on their relationship to the fetoplacental unit. The decidua basalis (or decidua placentalis) lies directly below the placenta. The decidua capsularis covers (encapsulates) the developing embryo as it grows and expands into the uterine cavity. The decidua parietalis (or decidua vera) lines the uterus remote from the placenta. A virtual space exists between the decidua capsularis and decidua parietalis until about 15–16 weeks of gestation, at which point these two tissues come together and fuse.

The decidua is the maternal tissue most intimately associated with the fetoplacental unit and serves a critical role as an endocrine and immunological organ. The process of implantation/placentation results in the formation of three decidual regions, which are anatomically and functionally distinct. This review focuses on that region that underlies the placenta, known as the decidua basalis (or decidua placentalis) (Figure 1).

The endometrium/decidua is a complex, dynamic, heterogeneous tissue made up of multiple cell types. Moreover, its cellular composition changes in a predictable fashion during the menstrual cycle and throughout the course of pregnancy in response to changes in systemic and local hormones. These cellular changes have been reviewed in detail elsewhere [17,31,32,33,34,35,36,37]. Importantly, the endometrium/decidua is rich in immune cells, particularly uNK cells and macrophages, which originate in the bone marrow and track selectively via the bloodstream to the uterine lining. In the first 20 weeks of pregnancy, uNK cells and macrophages play a critical role in mediating the process of spiral artery transformation by inducing initial structural changes, secreting a number of cytokines and chemokines, and promoting the actions of EVCTs [38,39,40]. They also protect against placental infection [41,42]. Another distinct and functionally important group of cells comprises the decidual stromal fibroblast cells (DSCs), which make up 10–30% of decidual cells in the first trimester and up to 60–70% of cells in term decidua (discussed below).

## 6. Endometrial Decidualization

Decidualization refers to the functional and morphological changes that occur within the endometrium to form the decidual lining into which the blastocyst implants. These changes include the recruitment of leukocytes and, importantly, the differentiation of endometrial stromal fibroblast cells (ESCs) into DSCs. It is the ability of ESCs to differentiate into this alternative cell state that appears to be the key element in the decidual transformation. DSCs are not simply modified ESCs; they are a distinct cell type resulting from terminal differentiation and the genetic reprogramming of ESCs. This reprogramming includes the downregulation of genes involved in the pro- inflammatory response and in resisting tissue invasion along with increased expression of genes that promote cellular proliferation, foster tolerance, and facilitate tissue invasion (discussed below). DSCs originated early in the stem lineage of placental mammals [43,44] and their evolution coincided precisely in evolutionary history with the appearance of invasive placentation [11,45].

## 7. Evolution of the Decidua

Decidualization is widespread among eutherian mammals and is perhaps best understood as a maternal solution to accommodate the invasive trophoblast. However, the presence of trophoblast within the uterine cavity does not always result in invasive placentation. In some placental mammals, most notably in hoofed animals such as the pig, placentation is superficial despite the fact that trophoblast cells retain the ability to invade ectopically [46]. Such species evolved an alternative and yet equally successful strategy to tolerate the presence of the hemi-allogeneic fetal allograft, namely maternal resistance to invasion, resulting in non-invasive placentation and the lack of endometrial decidualization. The fact that different mammalian viviparous species have evolved alternative solutions to the challenge of invading trophoblast highlights the important role that the maternal–fetal interaction plays in determining the resulting pregnancy phenotype, both across and within species. The functional importance of decidualization in human pregnancy is incompletely understood, but it appears to play a critical role in facilitating the active embedding of the conceptus [47], in the negative selection of nonviable embryos [48], in determining the optimal window of implantation [49,50], and in uterine hemostasis [51,52]. Proper decidualization controls conception and the course of pregnancy and is a critical determinant of pregnancy success in humans [2]. In non-menstruating species, the embryo controls this process by delaying implantation [53].

## 8. Timing of Decidualization

In most mammals that exhibit decidualization, the uterine reaction that transforms the endometrium into decidua is triggered by the arrival of the blastocyst. In contrast, the endometrium in humans, anthropoid primates, and a few non-primate species (including several species of bats, elephant shrews, and the spiny mouse [54,55]) undergoes decidualization extemporaneously in every menstrual cycle and not as a reaction to the presence of a blastocyst. In the absence of a conceptus, decidualization in humans ends with shedding of the upper layer of the decidualized endometrium (i.e., menstruation), a process that is triggered by programmed progesterone withdrawal at the end of the luteal phase. This cyclic decidualization is notably more complex than simple epithelial changes during the estrous cycle, which can be observed in the reproductive tract of most placental mammals. The teleological advantage of cyclic decidualization remains unclear, but it is interesting to note that all species with cyclic decidualization share a particularly invasive type of hemochorial placentation as well as a long gestation [9].

## 9. Master Regulators of Decidualization

**Figure 2:**
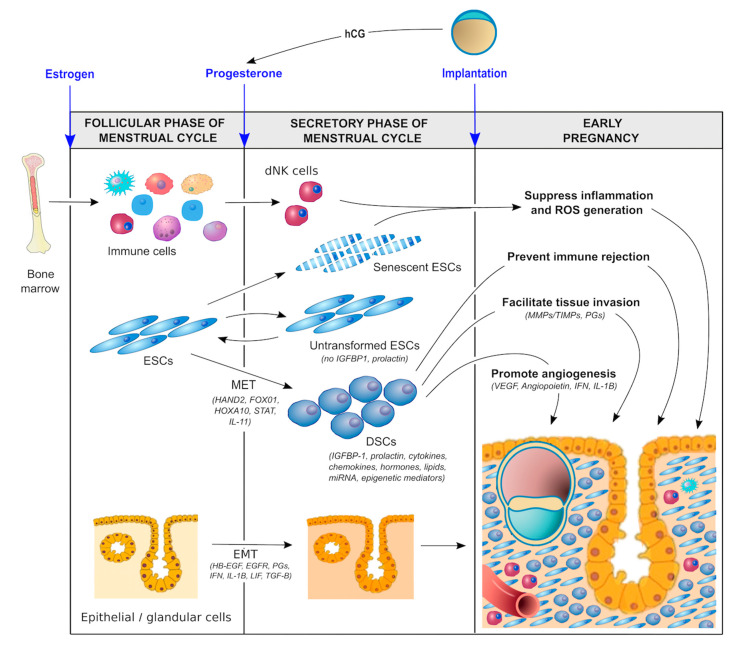
Molecular pathways involved in decidualization.

In order to promote optimal implantation/placentation and a healthy pregnancy, the endometrium must be optimally primed hormonally, biochemically, and immunologically during the luteal phase of the menstrual cycle (Figure 2).

- Hormonal factors. During the follicular phase of the menstrual cycle, estrogen production by ovarian granulosa cells causes the endometrium to proliferate and thicken. The major driver of decidualization is progesterone, which is produced by the corpus luteum of the ovary following ovulation. In the absence of a conceptus, the corpus luteum is programmed to regress in 14 days, resulting in systemic progesterone withdrawal and menstruation. In the presence of a pregnancy, production of human chorionic gonadotropin (hCG) by trophoblast cells prevents luteolysis, thereby maintaining progesterone production until the placenta takes over this functionality at 5–7 weeks of gestation [56]. Moreover, the local production of hormones such as relaxin and corticotropin-releasing hormone (CRH) in response to the hCG surge establishes an autocrine/paracrine regulatory loop to enhance intracellular cAMP levels in ESCs, promote decidualization, and support implantation and early pregnancy [57,58,59].
- Biochemical factors. There is increasing evidence to suggest that biochemical/metabolic factors are important in decidualization. For example, lipid mediators such as lysophosphatidic acid (LPA) are produced by uterine epithelium [60] and regulate heparin-binding epidermal growth factor (HB-EGF) [61] and epidermal growth factor receptor (EGFR) signaling as well as cyclooxygenase 2 (COX2) [62] and thereby prostaglandin E2 (PGE2) production, which together with interferon-γ control the spatial decidualization of ESCs [63,64]. Other autocrine/ paracrine factors—including interleukins, such as IL-1β, IL-11, and leukemia inhibitory factor (LIF) [65,66,67,68,69] as well as transforming growth factor-beta (TGF-β superfamily members such as activin, TGF-β1, bone morphogenesis protein 2 (BMP2), and left–right determination factor 2 (LEFTY2) [70,71,72,73]—also appear to be important in sustaining the decidualization process, promoting cAMP and extracellular matrix (ECM) signaling, regulating angiogenesis, and supporting embryo implantation. Glucose also serves as a metabolic signal for decidualization, providing a link between glycemic control and cellular oxidative stress (discussed below).
- Immunological factors. The importance of the immunological priming of the endometrium is becoming increasingly apparent. While this is driven, in part, by intrinsic factors, including a range of endocrine and autocrine/paracrine signals [3,17,35], extrinsic factors are likely also involved. One such factor is exposure to seminal fluid both prior to and around the time of implantation [74,75,76]. Interestingly, this exposure does not have to be local. Exposure to paternal antigen via nonvaginal routes can also prime the endometrium immunologically [77]. Although the mechanism responsible for this priming effect is not clear, seminal fluid contains soluble and exosome-borne signaling agents that promote leukocyte recruitment and generation of regulatory T cells (Treg cells) which suppress inflammation, promote vascular adaptation, and foster tolerance towards fetal antigens [78]. This mechanism could shed light on a number of well-recognized risk factors for the ‘great obstetrical syndromes’ that have thus far defied explanation. Why is it that nulliparity, young maternal age, IVF conception, the use of donor sperm, the short length of cohabitation, short inter-pregnancy interval, and the use of barrier contraception are risk factors for conditions such as preeclampsia and PTB? Could the common factor be a lack of exposure to protective seminal fluid? Recent data suggest that intercourse during IVF treatment cycles improves implantation success and pregnancy health [79], which is consistent with the hypothesis that exposure to seminal fluid promotes healthy decidualization and implantation.

## 10. Molecular Regulation of Decidualization

Proper decidualization is a critical determinant of pregnancy success. The endometrium must be optimally primed prior to and shortly after the arrival of the blastocyst (Figure 2). In addition to the aforementioned maternal hormonal, metabolic, and immunological factors, a number of other local factors are involved (discussed below). Some of these local factors are of embryonic origin, such as lactate, relaxin, CRH, and hCG [57,58,59,80,81,82,83], although a detailed discussion of these embryo-derived factors in perpetuating the process of decidualization is beyond the scope of this review.

**Figure 3:**
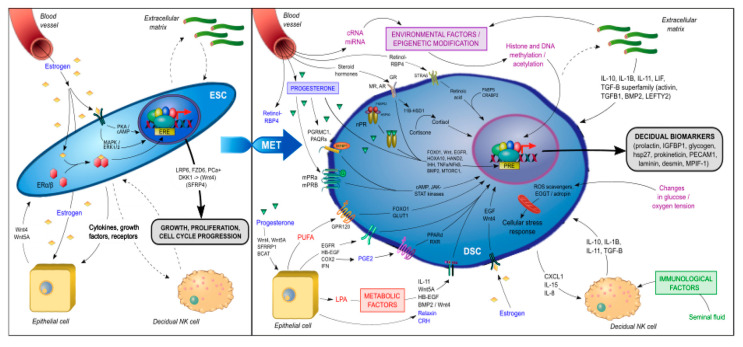
Signal transduction pathways involved in the genetic reprogramming and terminal differentiation of endometrial stromal fibroblast cells (ESCs) to decidual stromal fibroblast cells (DSCs).

During decidualization, differentiating ESCs carry a molecular signature of mesenchymal–epithelial transition (MET) as they are reprogrammed to become DSCs with widespread changes in gene expression, including the induction of such genes as HOXA10, HOXA11, FOXO1, WNT4, IGFBP1, and prolactin (PRL) [84,85,86,87]. Many of these are known upstream regulators of genes critical for implantation and placental development [17,18]. The signal transduction pathways involved in the genetic reprogramming and terminal differentiation of ESCs into DSCs (summarized in Figure 3) can be classified into several categories:

- Genomic progesterone signaling pathways mediated by the nuclear progesterone receptor (nPGR). nPGR is the dominant member of the 3-ketosteroid nuclear receptor family that responds to progesterone and cyclic AMP/protein kinase A (cAMP/PKA) signaling during decidualization [88,89]. A recent study that employed both RNA-sequencing and PGR chromatin-immunoprecipitation (ChIP)-sequencing of endometrium during the window of implantation showed that the PGR signaling network is made up of multiple different classical signaling pathways and involves numerous downstream regulators [90], including Indian hedgehog (IHH) [91], heart and neural crest derivatives-expressed (HAND2) [92], transcription factors Forkhead Box O1 (FOXO1) [93], SPR-related HMG-box gene 17 (SOX17) [94] and signal transducers and activators of transcription (STAT) transcription factor members (STAT1, STAT3, STAT5) [95], Notch signaling [96], insulin receptor substrate 2 (IRS2) [97], BMP2 and WNT signaling [72], HOXA10 [98], CCAAT/enhancer-binding protein β (CEBPB) [99], EGFR [100], mammalian target of rapamycin complex 1 (MTORC1) [101], and the tumor necrosis factor alpha-nuclear factor kappa-light-chain-enhancer of activated B cells’ (TNFα/NFκβ) pathway [102]. These pathways play an important role in the embryo–uterine, epithelial–stromal, and stromal–immune cell crosstalk that occurs in the peri-implantation period and is responsible for such functions as EMT, insulin resistance, focal adhesion, trophoblast invasion, regulation of the complement and coagulation cascade, cytokine-cytokine receptor interactions, xenobiotics metabolism, inflammatory response, ECM receptor interaction, angiogenesis and vasculature development, apoptosis, cytoskeleton remodeling, and the secretion of glycogen and other decidualization markers, such as PRL and insulin-like binding factor (IGFBP1). In a proteome and secretome screening study of in vitro decidualized ESCs, Garrido-Gomez et al. [103] reported that, in addition to PRL and IGFBP1, a number of other secreted decidualization markers might be involved in the attendant angiogenesis, including platelet/endothelial cell adhesion molecule-1 (PECAM-1) and myeloid progenitor inhibitory factor-1 (MPIF-1). In another study of 23 secreted factors derived from primary ESCs prior to ART, coordinated and synchronized changes in the secretome were associated with successful implantation, whereas cultures from the failed implantation group typically demonstrated a disordered secretome profile [104].

- Non-genomic progesterone functions not mediated by nPGR. Recent studies have revealed the presence of membrane-associated putative progesterone-binding proteins, such as PGR membrane component 1 and 2 (PGRMC1, PGRMC2) [105] and progestin and adiponectin receptors (PAQRs) [106], in cycling endometrium and pregnancy tissues that can rapidly activate downstream signal transduction pathways to mediate non-genomic functions of progesterone, including interacting with PGR [107] and other steroid receptors [108], regulating endometrial receptivity [109], and triggering and promoting parturition [110,111]. The functional importance of these membrane-associated proteins in decidualization remains unknown. However, in addition to nPGR, other members of the 3-keto-steroid nuclear receptor family—such as glucocorticoid receptor (GR), mineralocorticoid receptor (MR), and androgen receptor (AR)—have also been found to play an important role in decidualization. In an in vitro decidualization model in which ESCs were induced with 8-bromo-cAMP (8-Br-cAMP) and medroxyprogesterone acetate (MPA), Cloke et al. demonstrated that AR regulated the expression of a distinct decidual gene network with a preponderance of upregulated genes being involved in cytoskeletal organization and cell motility and repressed genes being involved in cell cycle regulation [112]. Moreover, Kuroda et al. reported that progesterone/cAMP induction of ESCs increased expression of the 11β-hydroxysteroid dehydrogenase type 1 (11β-HSD1) enzyme, which converts inert cortisone to active cortisol and thus contributed to the metabolic regulation in decidualizing ESCs [113]. Taken together, these data suggest that the decidualization of ESCs involves the integration of multiple nonredundant signaling networks in response to progesterone stimulation.
- Metabolic regulators. The increased 11β-HSD1 expression and activity associated with ESC decidualization leads to a decrease in GR and reciprocal increase in MR expression [113]. The upregulation of MR-dependent genes, in turn, affects lipid droplet biogenesis and retinoid metabolism. For example, 11β-HSD1 upregulates dehydrogenase/reductase 3 (DHRS3) expression, which promotes retinol storage in lipid droplets [113]. Retinoic acid (RA) is essential in the maintenance of pregnancy and its metabolism is tightly controlled at the maternal–fetal interface [114]. The decidualization of ESCs increases the expression of retinol-binding protein 4 (RBP4) and cytochrome P450 26A1 (CYP26A1) involved in RA metabolism and downregulates the expression of the pro-apoptotic RA nuclear receptor (RAR) [115]. The lipid mediator LPA also regulates EGFR signaling, COX2 expression, and prostaglandin signaling for the spatial decidualization of ESCs [63,64]. COX2 in turn activates uterine peroxisome proliferator-activated receptor-delta (PPAR-δ) and retinoid X receptor (RXR), which are critical regulators of decidualization and implantation [116]. Omega-3 polyunsaturated fatty acids have been shown in numerous animal and clinical studies to be beneficial for pregnancy outcome [117]. The receptor GPR120, a member of the rhodopsin family of G protein-coupled receptors, mediates potent anti-inflammatory and insulin- sensitizing effects [118]. Huang et al. showed that GPR120 could promote decidualization by upregulating FOXO1 and glucose transporter-1 (GLUT1) expression, glucose uptake, and pentose-phosphate pathway activation in ESCs [119].

Decidualization results in vascular remodeling with fluctuations in oxygen tension and the generation of reactive oxygen species (ROS). DSCs are programmed to resist a range of cellular stress signals to maintain the integrity of the feto–maternal interface and survival of the conceptus. Several molecular mechanisms have been implicated, including the inhibition of stress pathways such as c-Jun N-terminal kinase [120], attenuated inositol trisphosphate signaling [121], resistance to microRNA-mediated gene silencing [122], and the upregulation of free radical scavengers [123]. Another implicated pathway involves O-GlcNAcylation, a post-translational modification that links glucose sensing to cellular stress resistance. Muter et al. reported that the upregulation of the glycosyltransferase enzyme, EGF domain-specific O-linked N-acetylglucosamine transferase (EOGT), in decidualizing ESCs is responsible for the N-acetyl-glucosamine modification of a number of secreted and membrane-associated proteins involved in glucose and fatty acid metabolism [124]. Finally, in a uterine-specific *p53*-ablation PTB mouse model, decreased mitochondrial β-oxidation and ATP-production led to changes in lipid signaling and premature senescence of the decidua with subsequent PTB and/or stillbirth [125,126]. The inhibition of mTORC1 activity using rapamycin in this *p53*^−/−^ murine model attenuated the premature decidual senescence and rescued the PTB phenotype [127].

- MicroRNA (miRNA) and epigenetic regulation. Using the miRNA profiling of ESC primary cultures before and after in vitro decidualization, Estella et al. reported an upregulation of 26 miRNAs and the downregulation of miR-96, miR-135b, miR-181 and miR-183 [128]. The addition of miR-96 and miR-135b in decidualizing ESCs decreased the expression of FOXO1 and HOXA10 as well as IGFBP-1 secretion [128]. In another study, Jimenez et al. reported that the upregulation of the miR-200 family during in vitro decidualization of ESCs correlated with the downregulation of IHH signaling and expression of the EMT regulator, ZEB1 [129]. Similar studies have demonstrated the functional importance also of miR-181a [130], miR-542-3p [131], and miR-194-3p [132] in decidualization. While individual miRNAs can regulate a range of target genes, there is growing evidence that endometrial cells undergo genome-wide chromatin remodeling for the access of transcription factors or epigenetic modifiers during decidualization [133,134]. In particular, the expression of the histone methyltransferase Enhancer of Zeste Homolog 2 (EZH2) appears to be reduced in endometrium beginning in the mid-secretory phase of the menstrual cycle and specifically in decidualizing ESCs [135]. The knockdown of *Ezh2* in decidualizing human ESCs resulted in reduced levels of trimethylated lysine 27 of histone 3 (H3K27me3), a repressive histone mark for silenced genes, in the proximal promoter regions of the *PRL* and *IGFBP1* genes, with a reciprocal enhancement of histone acetylation and concomitant higher expression of these two gene products [136]. A recent combined H3K27me3 ChIP-Seq and RNA-Seq analysis of mouse decidual cells harvested at different gestation stages confirmed the H3K27me3-induced transcriptional silencing of target genes that specifically suppress inflammation and contractile function in early gestation. In late gestation, genome-wide H3K27me3 demethylation was observed, thereby allowing de-repression and target gene upregulation to lead to the onset of labor [136]. Moreover, the pharmacological inhibition of H23K27 demethylation was able to inhibit labor and delivery while maintaining pup viability in a PTB murine model [136], thereby demonstrating the functional importance of this molecular mechanism. These data are consistent with the hypothesis that parturition in humans is nothing more than a delayed menstruation [11]. Although intriguing, it should be noted that the function of EZH2 and genome-wide chromatin remodeling in the process of human decidualization and implantation remains unclear. Additional studies are needed to further investigate these epigenetic regulatory mechanisms within the various uterine compartments and their association with pregnancy outcome.

## 11. Decidualization Resistance and Pregnancy Complications

“Decidualization resistance” refers to the inability of the maternal compartment to undergo these decidualization changes leading to aberrations in implantation/placentation and adverse pregnancy outcome. The hypothesis that pregnancy health is shaped prior to implantation/ placentation and is determined by the health of the decidua may shed light on the reproductive phenotype of a number of important clinical disorders.

- **Preeclampsia** is a pregnancy-specific disorder characterized by new-onset hypertension and maternal end-organ damage after 20 weeks’ gestation that complicates 5–7% of all pregnancies. The pathologic hallmark is shallow trophoblast invasion and a failure of spiral artery remodeling. The current model posits that the primary cause of this suboptimal endovascular invasion is a failure of the decidua to tolerate and/or facilitate trophoblast invasion. In support of this hypothesis, Garrido-Gomez et al. identified a transcriptomic fingerprint characterizing a decidualization defect in the endometrium of women with a history of severe preeclampsia that is linked to impaired cytotrophoblast invasion. Moreover, this defect was detected at the time of delivery and persisted for years thereafter [137]. The decidualization of ESCs is mediated, at least in part, by annexin A2 (ANXA2) and the maternal deficiency of the *ANXA2* gene contributes to shallow decidual invasion by placental cytotrophoblast cells [138]. These findings highlight the maternal contribution to the pathogenesis of severe preeclampsia.
- **Recurrentpregnancy loss**, defined as 3 or more consecutive miscarriages, is a condition experienced by 1–2% of all couples. The cause is poorly understood. It is widely attributed to either repeated chromosomal instability in the conceptus or ill-defined uterine factors. Recent studies suggest that such women have impaired cyclic decidualization that predisposes to pregnancy failure by disrupting the maternal response to hormonal signaling leading to the dysregulation of decidualization markers, including, among others, prolactin, prokineticin, and the genes *DIO2* and *SCARA5* [139,140].
- A similar mechanism may account for the increased risk of adverse pregnancy events in women with poorly controlled **pregestational** **diabetes**. In such women, strict glycemic control around the time of conception has been shown to reduce rates of miscarriage and birth defects (diabetic embryology) and to improve overall pregnancy outcome [141].

## 12. Prevention of Pregnancy Complications

To date, interventions to prevent late-onset pregnancy complications have been largely unsuccessful. There is currently no effective intervention to prevent GDM, FGR, or stillbirth. Efforts to prevent PTB have been similarly disappointing. Despite initial enthusiasm regarding the use of 17α-hydroxyprogesterone caproate (17P) supplementation to prevent PTB in patients at high risk due to one or more prior unexplained spontaneous PTBs based on a single clinical trial [142], follow-up studies have failed to show significant benefit [143]. If there exist a subset of women who do benefit from 17P supplementation—and it is not clear that there is—the mode of action is most likely through a direct anti-inflammatory effect of progesterone on the decidua rather than by supporting suboptimal circulating progesterone concentrations [111,144]. A plausible explanation for the extent of this failure is to be found in the current model, which posits that the foundation for adverse pregnancy events is laid down before pregnancy with abnormalities in endometrial decidualization. Once the pregnancy is established, it is too late to intervene effectively.

The one possible exception to our inability to prevent late pregnancy complications is low-dose aspirin prophylaxis to prevent preeclampsia in patients at high risk [145]. Aspirin is a nonselective inhibitor of COX enzyme, which suppresses the production of the primary pro-inflammatory mediators (PGF2α and PGE2) in the maternal decidua, thereby promoting immune tolerance and facilitating trophoblast invasion. However, it needs to be given early in pregnancy around the time of the second wave of trophoblast invasion and the protective effect is limited.

## 13. Future Direction

The notion that the health of a pregnancy is shaped by the success of endometrial decidualization and predates the establishment of the pregnancy may explain the mechanism by which pre-pregnancy lifestyle interventions—such as weight loss, strict glycemic control, and preconception folic acid supplementation [146]—improve pregnancy outcomes, namely by improving decidualization. Once the molecular pathways responsible for optimal decidualization have been characterized, existing drugs can be tested or orphan drugs repurposed and their effect on decidualization studied both in vitro (by sampling the endometrium using a variety of minimally invasive gynecological procedures or isolating decidualized cells from menstrual blood) and in vivo in nonpregnant menstrual cycles. The role of the reproductive tract microbiome in decidualization/implantation is not well understood and is another potential avenue for intervention. This approach provides an exciting opportunity for future innovation since any such treatment would be initiated and likely even completed before the pregnancy is established.

## 14. Conclusions

Although both a viable blastocyst and appropriately primed endometrium are necessary for successful implantation/placentation, we posit that the optimal priming (decidualization) of the endometrium is the most critical determinant of pregnancy success. Decidualization involves the recruitment of leukocytes and, critically, the genetic reprogramming of ESCs to DSCs that suppress inflammation, promote vascular adaptation, and foster tolerance to fetal antigens. Since decidualization in humans occurs during every menstrual cycle in the absence of the conceptus, pregnancy health is determined even before the blastocyst arrives. A better understanding of the molecular and cellular mechanisms responsible for endometrial decidualization will improve our understanding of the factors influencing pregnancy success and provide opportunities for novel approaches to diagnosis and treatment.

## Abbreviations

11β-HSD1	11β-hydroxysteroid dehydrogenase type 1
AR	androgen receptor
BCAT	branched chain amino acid transferase
BMP2	bone morphogenesis protein 2
COX2	cyclooxygenase 2
CRABP2	cellular retinoic acid binding protein 2
CRH	corticotropin-releasing hormone
CXCL1	C-X-C motif chemokine ligand 2
DKK1	Dickkopf WNT signaling pathway inhibitor 1
DSC	decidual stromal cell
EGF	epidermal growth factor
EGFR	epidermal growth factor receptor
EOGT	EGF domain specific O-linked N-acetylglucosamine transferase
ERK	extracellular- signal-regulated kinase
ESC	endometrial stromal cell
FABP5	fatty acid binding protein 5
FOXO1	transcription factors Forkhead Box O1
FZD5	Frizzled 5
GR	glucocorticoid receptor
HAND2	heart and neural crest derivatives-expressed 2
HB-EGF	heparin binding EGF-like growth factor
HSP	heat shock protein
IFN	interferon
IGFBP1	insulin-like growth factor binding protein 1
IL-1β	interleukin-1β
IHH	Indian hedgehog
LEFTY2	left-right determination factor 2
LIF	leukemia inhibitory factor
LPA	lysophosphatidic acid
LRP6	LDL receptor related protein 6
MAPK	mitogen activated protein kinase
MET	mesenchymal-epithelial transition
miRNA	microRNA
MPIF-1	myeloid progenitor inhibitory factor-1
mPR	membrane progesterone receptor
MR	mineralocorticoid receptor
MTORC1	mammalian target of rapamycin complex 1
NK cells	natural killer cells
nPR	nuclear progesterone receptor
PAQRs	progestin and adiponectin receptors
PCa+	prostate cancer a protein
PECAM-1	platelet-endothelial cell adhesion molecule-1
PGE2	prostaglandin E2
PGRMC1	PGR membrane component 1
PPAR-δ	peroxisome proliferator-activated receptor-delta
PUFA	polyunsaturated fatty acids
RBP4	retinol-binding protein 4
ROS	reactive oxygen species
RXR	retinoid X receptor
SFRP4	secreted frizzled-related protein 4
TGF-β	transforming growth factor-β
TNFα/NFκβ	tumor necrosis factor alpha-nuclear factor kappa-light-chain-enhancer of activated B cells
DC	dendritic cells
dNK	decidual natural killer cells
DSCs	decidual stromal cells
EGFR	epidermal growth factor receptor
ESCs	endometrial stromal cells
HB-EGF	heparin binding EGF-like growth factor
EMT	epithelial-mesenchymal transition
hCG	human chorionic gonado-tropin
IGFBP1	isulin-like growth factor binding protein 1
IFN	interferon
IL-1β	interleukin-1β
ROS	reactive oxygen species
LIF	leukemia inhibitory factor
MET	mesenchymal-epithelial transition
miRNA	microRNA
MMP	matrix metalloproteinase
PGS	prostaglandins
TGF-β	transforming growth factor-β
TIMP	tissue inhibitor of meatalloproteinase
VEGF	vascular endothelial growth factor
